# What Youth Write About and Seek in an Anonymized Online Peer Support Forum: Insights from India

**DOI:** 10.3390/ijerph23030389

**Published:** 2026-03-18

**Authors:** Ravikesh Tripathi, Abhishek Karishiddimath, Pramita Sengupta, Khushboo Khatri, Lakshmisree KV, Jomy T. Jose, Athulya Elsa Idicula, TK Srikanth, Seema Mehrotra

**Affiliations:** 1 Department of Clinical Psychology, National Institute of Mental Health and Neurosciences, Bengaluru 560029, Indiaabhishek.karishiddimath@gmail.com (A.K.); athulyaelsaidicula@gmail.com (A.E.I.); 2 E-Health Research Centre, International Institute of Information Technology (IIIT), Bangalore 560100, India

**Keywords:** peer support, peer support forum, online peer support forum, digital peer support, youth mental health

## Abstract

**Highlights:**

**Public health relevance—How does this work relate to a public health issue?**

- High prevalence of psychological distress in youth, combined with preference for self-reliance and informal support and inclination towards digital platforms, highlights the potential of anonymized online peer support forums as one of the accessible avenues for supporting youth mental health.

**Public health significance—Why is this work of significance to public health?**

- Youth posts on the forum reflected diverse personal, relational, social, and achievement-related concerns, often accompanied by intense negative emotional disclosures.
- Venting distress, seeking peer suggestions, sharing perspectives, meaning-making, and seeking validation emerged as the key purposes of posting on the forum. These findings underscore the scope and utility of such forums.

**Public health implications—What are the key implications or messages for practitioners, policy makers and/or researchers in public health?**

- Such anonymous, moderated forums can offer accessible, low-intensity support for youth in distress. It can be integrated into mental health promotion activities to deliver high-quality, responsive care.
- Future studies should examine how such platforms can foster professional help-seeking when needed, leveraging peer norms and behavioral nudges.

**Abstract:**

A high prevalence of psychological distress and unmet mental health needs among youth, combined with a preference for self-reliance and informal support (e.g., peers), poses a major public health challenge. Growing reliance on digital platforms highlights the potential of anonymized online peer support forums as accessible first-line avenues of support. In India, research on peer support interventions remains scarce. This study aimed to identify the concerns for which Indian youth engage in an anonymized, moderated online peer support forum, as well as the purposes for posting. A retrospective qualitative design was employed, analyzing all 137 posts of 124 unique users received between February 2024 and October 2025 on the forum using hybrid thematic analysis. Findings revealed that user posts encompassed diverse concerns across personal, relational, social, and achievement domains. Feeling states emerged as the most prominent theme, frequently co-occurring with other themes, followed by self-related concerns. Several posts explicitly mentioned mental health concerns such as depression and social anxiety, often without mention of professional help-seeking suggestions. Family-related issues, romantic relationship concerns, academic pressures, social comparisons, and unmet needs for approval were some of the other themes that emerged. Shifting from content to motivations for posting, the analysis identified purposes such as venting distress, seeking suggestions, sharing reflections, engaging in meaning-making, and seeking reassurance or validation. Future work needs to examine whether such forums can function not only as spaces for strengthening self-help and peer support processes but also as avenues for improving professional help-seeking through normalization and encouragement of the same when appropriate.

## 1. Introduction

Youth and young adults are recognized as a vulnerable group as far as mental health is concerned. For example, nearly one-third of first-year university students across 19 universities in eight countries screened positive for at least one common mental disorder, including anxiety, mood, or substance use disorders [1]. Beyond diagnosable conditions, subthreshold symptoms are widespread—for example, depressive symptoms affect up to 29% of youth and can impair functioning, social activity, and academic performance, while increasing the risk of major depressive disorder and suicide attempts [2]. Similarly, an international study of undergraduates from 12 countries spanning Europe, Asia, the Western Pacific, and North and Latin America reported that 48% of participants experienced clinically significant depressive symptoms [3].

India, home to one of the largest youth populations globally [4], reflects similar concerns. In a survey of Indian college students across nine states, 33.6% reported moderate to severe depressive symptoms and 23.2% reported moderate to severe anxiety symptoms [5], alongside reluctance to seek help due to poor awareness, normalization of distress, stigma, fear of judgment, and access barriers [6,7,8].

Empirical evidence shows that youth often prefer informal support from peers, friends, and family over formal services [6,9,10]. This becomes even more relevant for collectivistic societies like India [11,12,13]. Emotional disclosure, or the social sharing of emotions, facilitates regulation and recovery by reducing rumination while stimulating supportive social interactions [14,15]. Online platforms extend this natural tendency for social sharing, connecting, venting, and validation [16,17,18].

Digitalization has transformed the mental health sector worldwide, opening new pathways for accessible, low-intensity interventions. Digital mental health tools and apps provide scalable, evidence-based support for individuals who may not otherwise have access to face-to-face treatment [19,20]. Youth and young adults, among the most active online populations [21], increasingly turn to digital platforms to seek information, share experiences, and manage everyday stressors [22,23,24]. These platforms are particularly appealing due to their accessibility, anonymity, cost-effectiveness, and reduced stigma [25,26].

Consistent with the World Health Organization’s stepped-care pyramid, low-intensity interventions such as self-help strategies and peer support are recommended for addressing milder concerns and providing scalable first-line support [27]. In this context, low-intensity, digitally mediated peer support can offer one of the promising avenues to bridge unmet needs to some extent, especially for concerns that may not require a clinical diagnosis but still affect wellbeing. Moreover, such online spaces can also encourage professional help-seeking for problems of higher severity.

Peer support, defined as “giving and receiving help founded on respect, shared responsibility, and mutual agreement of what is helpful” [28], is increasingly being delivered through technology. Digital peer support includes peer-to-peer networks on social media, smartphone-supported interventions, and synchronous/asynchronous platforms [29]. Evidence shows that such interventions can enhance selfhood, compassion, and mindfulness, while lowering depressive and anxiety symptoms among emerging adults [30]. Online communities also provide safe spaces for youth who feel unsupported offline or fear stigma when disclosing sensitive issues [31,32,33,34]. Qualitative studies highlight that young people use these forums for venting, journaling, and “getting things off their chest” when disclosure feels difficult in real life [35]. They exchange emotional and informational support, coping strategies, and identity management insights [36]. Topics discussed range from anxiety, depression, panic attacks, eating disorders, suicide, and self-harm to interpersonal relationships, family issues, loneliness, bullying, school stress, identity, and sexuality [37]. Online peer support forums provide a safe space for emotional catharsis and may improve mental health outcomes, particularly when well-regulated and moderated [38]. Structured moderation enhances safety and ethical accountability compared to unsupervised platforms, with youth services such as Kooth demonstrating positive wellbeing and help-seeking outcomes [39,40,41,42]. Additionally, anonymity in digital settings facilitates greater self-disclosure and perceived relational safety among young users [43,44].

In India, research on peer support interventions in general remains scarce [45]. A preliminary Indian study found that 63.8% of young adults expressed interest in seeking support via online peer support forums for emotional and informational needs, demonstrating receptivity to moderated platforms with trained peer volunteers [18]. Another study described the development of an online peer support forum, associated challenges, and observed themes of posts spanning relationships, academics, and mental health concerns [46]. These findings underscore the potential of culturally sensitive, moderated peer support interventions in India.

Studying the nature and purpose of youth engagement in online peer support platforms can yield valuable insights into unmet mental health and wellbeing needs, the training required for peer support providers, and strategies to enhance the quality of support and resources offered. Such understanding can inform approaches to strengthen coping with distress, promote self-help, and encourage pathways to appropriate help-seeking [47]. Against this backdrop, the present study aimed to identify the concerns for which Indian youth engaged in an anonymized, moderated online peer support forum, as well as the purposes for posting, as reflected in their posts.

## 2. Methods

The study forms part of a larger research project reviewed and approved by the Institute’s Ethics Committee (NIMHANS/45th IEC (BEH.SC.DIV.)/2024).

### 2.1. Characteristics of the Peer Support Forum

The data used in the present study came from an online peer support forum (Let’s Talk Life) that is meant to provide space for Indian youth to voice concerns, doubts, or life-challenges that relate to their mental wellbeing and receive basic psychological support in an asynchronous mode. This forum allows registered users to post anonymously. No personally identifiable information is published, and the posts are publicly accessible to any visitor of the platform. All the responses to posts are drafted by trained volunteers and are posted after moderation by a team of mental health professionals. The forum informs the users that it is most suitable for Indian youth, whether college-going or working, within 18 to 25 years of age. However, it does not restrict older youth from registering as users.

The forum operates within a structured ethical and safety framework. Peer volunteers undergo extensive training, including vignette-based exercises and structured guidance on appropriate responding (e.g., dos and don’ts). To minimize unintended harm, the platform does not permit back-and-forth conversations or user-to-user interaction, although users may express solidarity through brief supportive reactions. Volunteers are trained to recognize risk markers and, in case of posts indicating suicidal or self-harm concerns, the responses, drafted in consultation with moderators, include appropriate local helpline information and encourage professional help-seeking. In certain instances, users may be encouraged to contact the moderators via email for referral-related information, if needed. The platform clearly states its scope and limitations (e.g., not a crisis service, not a substitute for professional care) within the “About” and “Terms and Conditions” sections and provides a dedicated resource page with credible mental health information and helpline contacts.

### 2.2. Ethical Aspects of Research on the Forum

With respect to research ethics, limited demographic information (e.g., email ID, age, gender) is collected at registration. However, these demographics of the users (age and gender) are not visible to either the volunteers or the moderators. This information was accessed from the backend only for the purpose of the study. As mentioned earlier, this forum allows registered users to post anonymously. Subsequently, no personally identifiable information is published, and the posts are publicly accessible to any visitor of the platform. The terms and conditions of the forum clearly state that forum-related anonymized content can be used for research. Furthermore, it is also indicated that participation in the forum constitutes agreement to the use of data under this term.

### 2.3. Eligibility Criteria for the Posts Analyzed

All posts (*n* = 137) received between February 2024 and October 2025 on this forum called “Let’s talk life” forum were analyzed using thematic analysis [48]. There were no preset eligibility criteria for selection of the posts and hence no exclusions.

### 2.4. Data Analysis

The 137 posts were contributed by 124 unique users; eight users submitted multiple posts, accounting for 21 posts in total (most posted twice, two posted thrice, and one posted five times). Each post was treated as an independent unit of analysis without drawing inferences from prior posts or responses. Although some users posted more than once, the content was not consistently linked and was typically spaced over time; therefore, posts were not considered interdependent for analytic purposes. However, for moderation and response drafting, prior posts and replies were reviewed to ensure continuity and avoid repetition in guidance.

The authors employed a pragmatic, codebook-style thematic analysis informed by Braun and Clarke’s six-phase framework, combining deductive parameters with inductive theme development [48]. The two parameters, i.e., concerns and purposes of posting, were redecided, as these anchor our study objective. When analyzing posts, the authors simultaneously coded the concerns and purpose of posting. An initial coding framework was developed based on the two analytic parameters. During iterative coding, new inductive codes were added, redundant codes were merged, and definitional clarity was refined across three rounds of team discussion. The codebook stabilized after analysis of approximately 25% of the 137 posts. Independent coding was used not to calculate inter-rater reliability but to enhance analytic breadth. Discrepancies were resolved through discussion, leading to refinement of the evolving codebook. Sufficiency was decided when no new categories emerged. A theme was defined as a meaningfully coherent category capturing a specific type of concern that recurred across multiple posts and demonstrated conceptual distinctiveness from other categories. Rather than restricting to a single theme, more than one theme was assigned to posts or codable segments of a post whenever a co-occurrence of themes was noted. Co-occurrence of themes was expected and hence treated as richness of users’ posts/experience rather than a problem. As far as the purpose of the posts was concerned, it was observed during the finalization of coding that a single theme appeared to suffice for coding the purpose of the posting in almost all instances. Excerpts presented in the paper were selected to illustrate diverse themes/sub-themes to the extent feasible. The excerpts are kept in their original form, but phrases like “You know”, “I guess”, etc., were removed for better readability.

The first three authors and the last author conducted the analysis. The first and last authors, with a doctorate in clinical psychology and over ten years of expertise in qualitative research and digital mental health, served as the mentors. Except for the second-to-last author, the rest of the authors helped in collating the posts and drafting the results. The diversity of researcher backgrounds in gender, qualifications, and experience facilitated triangulation, thereby enhancing the rigor of the analysis.

## 3. Results

Thematic analysis of the forum posts revealed 19 distinct themes, reflecting a varied spectrum of concerns spanning personal, relational, emotional, and social domains among users aged 18 to 34, with a slight predominance of females (56%). Seven of these themes included subthemes, while the remainder were standalone themes.

The following subsection describes in detail the themes with subthemes regarding the concerns reflected in the posts, with an overview presented in Table 1. The varied purposes underlying users’ posts are discussed separately in a later section.

### 3.1. Theme 1: Family

Concern related to family emerged as a theme. Various family issues such as sibling comparison, family conflict, wish to escape, role burden (non-normative), lack of family support, and family pressure were reflected in the users’ posts.

Users’ posts reflected how constant conflicts and disagreements within their family contributed to an uncongenial home environment, often leading to emotional withdrawal and desire to avoid or escape family spaces, as well as long-term psychological impacts such as heightened anxiety, difficulty in concentration, etc.

One user described their experiences with constant comparison and how it invalidated their individuality, *“…My parents have an annoying habit of constantly telling me to be like him. It’s as if they want me to be his carbon copy, not myself. The things I see as my strengths, like my compassion, they see as a weakness and label me oversensitive. The comparison is exhausting.”* (Post 64, Prefer not to say, 24 years).

Users also expressed the distress experienced when carrying the roles and responsibilities at home, particularly financial and caregiving-related. As a user shared, *“I have a lot of responsibilities, where as I’m only 18 years old where I earn money and also study, but day by day I’m exhausted with all these roles I need a break but If I don’t work then there is no one left out who will pay my fees and there is no one to earn money too…”* (Post 24, Female, 18).

These stressors were further exacerbated by the family’s expectation of the user to succeed, resulting in internalized pressure to “do more” or “prove themselves”, often accompanied by constant feelings of insufficiency. To quote one post, *“…why do you constantly feel the pressure to prove them wrong or the pressure that you are not doing enough and for once, how do I feel normal for once without all of this badgering in my head. I always wake up with this pressure to just push harder than yesterday. Yet, it never seems to be enough for my family…”* (Post 121, Female, 18 years).

### 3.2. Theme 2: Romantic Relationship

The theme Romantic Relationship Concerns reflected users’ emotional distress and uncertainty arising from romantic relationships. These sub-themes were ambivalence, preoccupation, coping with breakup, and jealousy.

A prominent aspect of this theme involved difficulty coping with breakups and relational loss. One user noted, *“the void is always there of a person who could be there and listen to me”* (Post 5, Female, 20 years).

Another pattern involved distress in ongoing relationships, marked by emotional invalidation and control. Users reported, *“I have been in a relationship for a year. 80% of the time we have cried for each other be it insecurity, control, misunderstandings,* etc. *I never blamed him for anything ever. He used to ask me to remove all my guy friends, he never complimented me”* (Post 29, Female, 23 years).

Having mixed feelings about relationships was also expressed in some of the posts. To quote, *“I have given him the comfort of being the perfect girlfriend but why do I feel he is not doing enough? He does communicate and gives me a sense of security but I do not feel loved, still. I feel lonely. He is patient and our mindsets are the same. But why do I still feel empty and forget how it feels to be loved?”* (Post 124).

### 3.3. Theme 3: Concerns Related to Significant Others

Posts within this theme involved expressions of worry experienced by users when witnessing people close/important to them in distress and the challenges they faced in supporting them. These sub-themes were concerns about the mental health of significant others, worries about not being helpful, challenges in supporting others.

One user expressed feelings of concern and worry and wanting to effectively support their long-distance partner who was experiencing distress. *“He has stopped eating properly, joking like he used to….talking to new people… he just stays alone. I fear his mental health will get worse and I won’t be able to help him”* (Post 62, Female, 23 years). This feeling was further exacerbated by the user’s expectation of herself, being a psychology student.

Another user noticed marked changes in the behavior of one of their peers, indicating distress/risk, but felt uncertain on how to approach/help them *“…but I’m not sure. I don’t know how to bring this up with him without sounding accusatory or judgmental…. I’m concerned for him!”* (Post 65, Prefer Not to Say, 24 years).

### 3.4. Theme 4: Feeling States

Difficulty navigating through different feeling states was recurrently noted in posts. Users mentioned varied feelings stemming from different life concerns. The sub-themes were: feeling betrayed in close relationships, feeling stressed and overwhelmed, feelings of uncertainty about future, feeling stuck, feeling worthless or not mattering, feeling hopelessness, death wishes, feeling helplessness, feeling a sense of desperation and urgency, feeling rejected, feeling numb, feeling lonely and needing companionship, feeling regret, senses of anger and unfairness, feeling like a burden, and feeling confused. This theme co-occurred the most along with other themes such as self-related concerns, romantic relationships, mental health concerns, academic challenges, etc. In this sense, it appeared like a cross-cutting dimension. However, the authors coded it as a theme as it depicts a wide range of feelings in the users’ posts. The description of feeling states often highlighted the intention of users regarding expressing or disclosing their experiences.

Feeling stressed and overwhelmed due to life circumstances was reflected in many posts. It was also associated with feeling “stuck” in the same situation. *“I am stressed, frustrated from this life where everything is happening against me. It’s not that I am not trying. I am trying very hard to focus but still not getting the results. I have many dreams to fulfill, but I am stuck in this phase.”* (Post 32, Male, 19 years).

Emotional pain from broken trust in relationships was expressed by some of the users. In their words, *“I thought I met a good person in my life then turned out to be a creep…I dumped him and i had a real good relationship with my male bestie and then we just had some argument and he said he needed some space a week later he’s telling me he doesn’t wanna talk I saw history repeating itself. I just can’t express how I feel right now. I just can’t trust guys anymore I feel”* (Post 134, Female, 19 years).

One of the users pointed out how their feeling of “mattering” was shaken. In their words, *“Hi, why do people think that I’m not important, even if I am with them they make me feel like I’m not important to them, they make fun of me…”* (Post 15, Female, 19 years).

Along similar lines, *“I sometimes feel alone because when I am trying to talk about something no one listens to what I am saying.”* (Post 21, Female, 20 years).

Another user stressed how they frequently experience self-doubt. *“During any small inconvenience, I feel timid, worthless and giving up on everything which feels like a burden mentally and feel the bare minimum is I can survive. I try to motivate myself but still I end up self-doubting.”* (Post 111, Female, 18 years).

The silent weight of feeling like a burden was also reported by some users.

*“I feel like I’m worthless, as if I’m of no use… Sometimes I feel like I’m a burden here.”* (Post 14, Female, 19 years).

The heaviness of feeling hopeless was also reflected in some of the posts.

*“I have gone through a lot of backlogs in my first sem. Despite the hard work I have done, nothing seems to work. I feel like a failure. I just feel hopeless.”* (Post 7, Female, 19 years).

Feeling helpless was also expressed by some of the users.

*“When cancer hit me and locked me behind closed doors i thought I could face it but sometimes the people behind closed doors just break you too hard and make you feel declined and unfit to be a part of this … I always wake up with this pressure to just push harder than yesterday. Yet, it never seems to be enough for my family. It just makes me sad that I probably will never live up to their expectations. I don’t want to live a life being fake to myself lying that I can do this. I’m helpless.”* (Post 121, Female, 18 years).

Feeling like time is running out and a sense of desperation and urgency was also found in some of the posts.

*“I am trying very hard to focus but I’m still not getting the results. I desperately want success now.”* (Post 32, Male, 19 years).

While users shared how they felt, some of them also showed their concerns about feeling “numb”, which troubled them.

*“…, why do I feel numb even if things around me are ok. Just why”* (Post 123).

### 3.5. Theme 5: Mental Health Concerns

One of the most reflected themes that emerged was “mental health concerns”. The subthemes were depression, anxiety, social anxiety, phobia, schizophrenia, ADHD, memory issues, digital addiction, pornography addiction, and trauma. Posts within this theme reflected how the users understood their own experiences surrounding psychological distress, with a few instances of self-diagnosis or professional diagnosis, along with narrating the interference these difficulties caused in their academic and interpersonal domains. These concerns were at times presented without any challenging life context, while in some other posts, these were described as either arising in the context of other themes or impacting various life domains (e.g., academic pressure, family dynamics), highlighting their multidimensionality.

Across the mental health concerns expressed on the forum, there are a variety of subthemes in terms of different kinds of symptoms (e.g., anxiety, social anxiety, depression, ADHD and schizophrenia, experiences with trauma), some of which were reportedly professionally diagnosed. Other concerns, such as digital overuse and addiction, with compulsive behavior impacting overall quality of life, were also highlighted. As one user shared, *“I am addicted to porn masturbation, smoking, alcohol, the internet and other things that I don’t know compulsive behaviour and other things to do, doing good in life, but addiction is killing me.”* (Post 127, Male, 27 years).

Anxiety-related concerns were prominent, particularly in academic and social settings. Multiple users shared experiences of nervousness and accompanying bodily manifestations that interfered with their daily functioning during examinations or other crucial deadlines. One user shared, *“In exam season I constantly feel nervous, anxious and will be unable to concentrate and study…”* (Post 112, Male, 18 years). Some users also reported experiencing social anxiety when speaking with or in front of people.

Several posts reflected the experiences of users with depressive symptoms characterized by feelings of loneliness, low moods, emotional numbness, loss of interest in previously enjoyable activities, concentration issues due to mood, unexplained tiredness, sleep disturbances, etc. Some users also expressed their struggles with making sense of their emotional reactions, their finances, and their inability to afford antidepressant medications, further adding to their strains.

A few posts reflected the lived experiences of users clinically diagnosed with mental health conditions like paranoid schizophrenia and ADHD. While these users demonstrated awareness about their symptoms, they also expressed the challenges they faced in their day-to-day functioning, *“I have found myself at the receiving end of symptoms of dissociation and disconnection. This happens quite frequently in the evenings and is quite scary for me. These are the biggest battles I wage on a near daily basis.”* (Post 52, Female, 20 years), a user diagnosed with a severe mental health concern, shared.

### 3.6. Theme 6: Self-Related Concerns

This theme captured instances wherein the content of posts revolved around users’ perceptions and evaluations about themselves, reflections on self-worth, self-perception, and internalized self-evaluations. The sub-themes were self-doubt, low self-confidence, shame, self-hatred, and self-criticality.

One user shared that during moments of self-doubt, they felt *“timid, worthless and giving up on everything”,* despite attempts to self-motivate, ultimately concluding that *“the bare minimum is I can survive”* (Post 111, Female, 18 years). This reflected a fragile sense of self-worth, where everyday challenges were interpreted as evidence of personal inadequacy.

Some users also reported a lack of self-confidence: *“I am from a small city and whenever a company comes for interviews, I feel low and not confident.”* (Post 43, Male, 22 years).

A sense of shame was also echoed in some of the posts. One of the users said, *“I hate it every time I breathe. Something is wrong with me. I should not be here, I am not good enough to have a life. I am ashamed of myself. A disgrace to this body. I hate myself.”* (Post 31, Male, 19 years).

One user expressed a self-critical stance and questioned whether they *“even deserve the friends I meet”* after realizing the impact of their words, expressing a belief that they were inherently “the problem” and undeserving of good experiences (Post 119, Female 19 years).

### 3.7. Theme 7: Concerns with Implementation and Planning

This theme captures the difficulty with implementation and planning across two sub-themes, e.g., time management and financial management.

Difficulties with time management, like managing distractions, adhering to routines, and completing tasks as planned, were reflected in the posts: *“I can’t seem to manage time effectively. I am unable to stick to a routine which makes me feel sad and disappointed in myself.”* (Post 55, Female, 25 years).

Users also expressed challenges they experienced in managing finances, sometimes linked to self-doubt and comparison with peers, *“Gaining financial independence is so important and I am scared, like what if that money I earn isn’t enough for myself… this might be easy for some but definitely not for me.”* (Post 103; Female, 19 years).

Posts under this theme occurred along with feeling stuck, stressed, overwhelmed, and self-doubting. Some posts co-occurred with other categories pertaining to role burden, self-doubt, low self-confidence, social comparison, academic pressure, efforts not leading to outcomes, and motivation management difficulties.

Additional standalone themes, i.e., themes without any sub-themes and their supporting excerpts, are presented in Table 2.

### 3.8. Purposes Underlying Posts

In addition to exploring the nature of concerns, the study sought to understand the underlying purposes reflected in user posts. The analysis revealed several distinct motivations:

- Seeking suggestions (*n* = 61): The majority of posts indicated that users were seeking specific advice or strategies to address their concerns. For example:
  ○ *“It’s difficult for me to manage my expenses due to the greed of wanting more. So can anyone give me some expense management tips so I can manage my greed in the proper way.”* (Post 12, Male, 21 years).
  ○ *“The structures and synthesis in pharmacy subjects are very difficult to memorize… So can anyone give me a wonderful tip to memorize all the synthesis and structure.”* (Post 13, Male, 22 years).
  ○ *“I’ve been struggling with anxiety lately, and they’re really starting to interfere with my daily life…. Does anyone have tips on how to cope when an anxiety attack hits? Thank you.”* (Post 58, Female, 25 years).

These excerpts illustrate users’ desire for actionable guidance and practical suggestions to address their ongoing challenges.

Some users expressed uncertainty about their situations and sought broader perspectives rather than specific advice. For instance:

  ○ *“Things are going okay, but I used to laugh more. Now I feel that fire within me die out each day. I am scared of the boring lifeless adult I might end up becoming. Should I aggressively just take some action and force myself to do what I would rather do? The thing is I am not even sure what that is anymore.”* (Post 128, Male, 25 years).

- Venting and emotional expression (*n* = 45): Many users utilized the forum as a space to vent and share their feelings, often about interpersonal difficulties or loneliness. For example:
  ○ *“I often feel people around me use me for their own deeds and don’t really care about mine. I do so much for people who don’t even try to reach out when I admit to them what I’m going through.”* (Post 75, Female, 19 years).
  ○ *“It is stressful that I cannot trust anyone…They are either busy or are not interested in listening to my problems or the situation I am going through. It’s not only about depressing or sad thoughts I cannot share happy incidents too. As a single child it makes me lonely at times.”* (Post 99, Female, 18 years).

These excerpts demonstrate the use of the forum for emotional release and the need for empathetic listening. Venting was also coded in some instances as the only implied purpose when no other purpose-based codes seemed to be evident.

- Sharing reflections and perspectives (*n* = 16): Some posts were characterized by users sharing their personal philosophies or reflections on life experiences. For example:
  ○ *“Being single means free from attachment, caring, tension, breakup…Happy for our self and caring for our self,..”* (Post 19, Female, 19 years).
  ○ *“Sometimes I feel like I may fail but still somewhere I feel I can do it. Failure is a first step in learning…… Believing in myself…… So that I can do anything.”* (Post 100, Female, 18 years).
  ○ *“As I am growing older, I look back on all the life choices I have made and sometimes I wonder if I made the right ones.”* (Post 70, Female, 28 years).

These reflections highlight users’ attempts to make sense of their experiences and foster self-understanding.

- Meaning-making (*n* = 10): A number of posts revealed users’ efforts to understand changes in themselves or their circumstances, often questioning the reasons behind their feelings or behaviors. For example:
  ○ *“I was very talkative… now a days I like to be alone, and it makes me better but this changes which was found by me just in a month but How?…”* (Post 18, Female, 19 years).

This excerpt illustrates the process of self-inquiry and the search for meaning in personal change.

- Seeking reassurance and validation (*n* = 5): Some users explicitly sought reassurance, affirmation, or validation of their worth and feelings. For example:
  ○ *“All I just wanna feel is to be loved, to be wanted, to be an important person in someone’s life. I don”t like hurting people…When I try to be honest people tell me that, I am rude. I thought maybe distancing myself from people would do good to them, but people told me I push others into depression. Am I really so bad? Don’t I deserve love for once?”* (Post 26, Female, 22 years).
  ○ *“Is it wrong to question your boyfriends when you are hurt even though it is repetitive? Is it considered nagging? I have given him the comfort of being the perfect girlfriend but why do I feel he is not doing enough?”* (Post 124).

These excerpts reflect the need for validation, acceptance and reassurance from peers.

We also attempted to arrive at a broad understanding of how the purposes of posting tended to co-occur with the nature of users’ concerns. “Venting Out” and “Seeking Reassurance” frequently co-occurred with “Feeling States”. “Seeking Suggestions” as a purpose often appeared alongside themes such as “Mental Health Concerns” and “Academic Pressure”. “Sharing Reflections and Perspectives” commonly co-occurred with “Reflection and Growth”. Efforts at “Meaning Making” were noted particularly in posts that also reflected “Confusion about Changes in Self or Mood” and “Feeling States”.

## 4. Discussion

The present study identified the concerns for which Indian youth engaged in an anonymized, moderated online peer support forum, as well as the purposes for posting, as reflected in their posts.

Findings from the study show that users’ posts on the peer support forum encompassed a wide range of concerns across personal, relational, social, and achievement domains. As a moderated, asynchronous platform, the LTL forum encourages youth and young adults to share life challenges and wellbeing concerns without focusing on any specific mental health issue or problem [42]. This broad orientation likely explains the wide range of concerns evident in the posts.

The content of posts revealed a broad spectrum of difficult emotions, including sadness, loneliness, disappointment, regret, guilt, anger, jealousy, and feelings of worthlessness, underscoring the role of such platforms in facilitating emotional disclosure. Anonymous support forums such as LTL can provide a safe space for discussing deeply personal experiences and difficult emotions, free from concerns about impression management or judgment within one’s social circle. Prior research has shown that youth expressions of emotion on social media predominantly highlight positive emotional displays [49]. In contrast, the posts on LTL were characterized by greater openness to expressions of negative affect, suggesting that the anonymity and supportive context of peer forums may encourage more candid sharing of distressing experiences.

Family emerged as an important theme in the posts, spanning sub-themes such as sibling comparisons, family pressures, and disturbed family environments impacting wellbeing. These narratives highlight the central role of family in the lives of young adults in an Indian context, which is characterized by interdependence, collectivist orientations, and the salience of family expectations in shaping identity and mental health outcomes [11,12,13].

Social comparisons, feelings of being misunderstood, unmet needs for social approval, and challenges in the context of romantic relationships were recurrent themes in the posts. These concerns align closely with the developmental tasks and psychosocial challenges faced by youth and young adults, particularly in contexts where identity formation, peer acceptance, and negotiation of intimate relationships are central to wellbeing. Social comparison and the quest for approval are critical in shaping self-concept and adjustment [50], and difficulties in close relationships and experiences of being misunderstood are common struggles during the transition to adulthood, often influencing mental health outcomes [51,52].

Several posts explicitly mentioned mental health concerns such as depression, social anxiety, bipolar disorder, and schizophrenia without any accompanying mention of ongoing engagement with professional services. Low rates of professional help-seeking are well documented in both global and Indian research, with peer norms shown to strongly influence youth willingness to seek care [6,9,10]. Social support and encouragement from significant others have consistently been identified as a critical facilitator, helping individuals overcome barriers to accessing professional services. Recent studies also highlight the growing use of technology-enabled approaches to promote help-seeking as a targeted intervention [47].

Shifting from the content of concerns to the motivations for posting, the analysis identified several overarching themes: venting, seeking suggestions, sharing reflections and perspectives, engaging in meaning-making, and seeking reassurance and validation. Qualitative studies suggest that young people often use online forums as spaces for venting, journaling, and “getting things off their chest” when disclosure feels difficult in offline contexts [37]. Within these interactions, they exchange emotional and informational support, coping strategies, and insights related to identity management [36]. Consistent with prior work, the present study also found evidence of both directional support (advice, guidance, and suggestion on coping strategies) and non-directional support seeking (venting, seeking reassurance, validation, and sharing reflections) [37].

Some posts reflected users’ ongoing efforts to make sense of difficult experiences and life challenges—a process commonly described as situational meaning. This involves attempts both to understand why certain events are occurring and to discern their personal significance. In the meaning-making literature, these efforts are often framed as searching for comprehensibility and searching for significance, both of which are recognized as central processes in adapting to adverse life circumstances [53,54].

In addition, a few posts reflected the meanings that users were constructing out of their struggles to understand life’s experiences. In most instances, these meanings were negative. These are likely to suggest a search for alternative perspectives or a desire to disclose distress and receive validation. Such expressions are consistent with research showing that meaning-making often emerges in contexts of distress and that articulating negative interpretations can function as an attempt to mobilize social support and reframe experiences [37,53,55,56,57].

In a few posts, meaning-making processes were also evident in terms of users reflecting on changes in their moods and selves and attempting to interpret their significance. Such sense-making reflects youths’ journeys in recognizing their concerns as potential problems requiring support. This aligns with Rickwood et al.’s [58] staged model of help-seeking, which emphasizes awareness and appraisal of problems as the initial step in the pathway to seeking help. Within this framework, the articulation of distress and efforts to make meaning of one’s experiences are integral to moving from the normalization of difficulties toward their problematization, thereby opening the possibility of professional help-seeking.

Research on youth perspectives indicates that online forums, while not a substitute for professional counselling, are seen to serve as valuable spaces where young people can access additional support. These forums are perceived as supportive environments that enable participants to ask questions, share advice, and foster a sense of connection, helping them feel less isolated and more engaged with others [23]. In addition, such forums may serve the purpose of enhancing help-seeking from professional sources [58].

Looking for suggestions was a common purpose noted in the posts in our study, but references to the need for professional help were infrequent. Viewing online support forums as a space that links youth to professional help has been observed to be one of the least-endorsed purposes by youth in an Indian study involving young adults [18]. On the other hand, soliciting general suggestions and advice, ideas on how to deal with a life situation, and factual information, in addition to receiving comfort and gaining new perspectives, were some of the most-endorsed functions of mental health support forums from the perspectives of youth [18]. In the present study, against the backdrop of significant disclosures about mental health concerns, reflections on personal changes and mood fluctuations, and descriptions of negative emotional states, this pattern suggests that posts were used primarily for expression, validation, and direct peer advice rather than for exploring whether or how to seek professional help. This, in turn, has implications for peer support providers in terms of how they encourage professional help-seeking when posts reveal concerns that may benefit from such intervention.

Youth frequently turn to the internet as a resource for understanding and addressing personal and health-related concerns [24]. In particular, young adults demonstrate a strong interest in technology-based tools and online communication, often relying on these platforms to access information and guidance regarding mental health issues [59]. Digital spaces also provide opportunities for young adults to exercise autonomy and seek support for complex and sensitive matters [59]. At the same time, young adults often normalize their distress, redefining what counts as “real” suffering by shifting their threshold toward extreme notions of normality [60]. Recognition and articulation of distress are only the first steps; individuals must also feel motivated to share their concerns with a potential source of support. In this process, friends and peers consistently emerge as the most likely and trusted sources of help when young adults experience mental health concerns such as depressive symptoms, underscoring the central role of peer networks in help-seeking pathways [9]. However, offline help-seeking is also known to be impeded by a number of barriers, such as the need to not burden others, stigma, and a preference for self-reliance [60]. These empirical findings point towards the role that online peer support forums, particularly anonymized forums, can play in offering opportunities to youth to bypass barriers to seeking offline informal help, particularly for emotional concerns that may not be easy to share with significant others.

The study had a few limitations. First, only a limited number of posts that were available during the study period were analyzed. Second, the LTL forum, by design, permits only a restricted number of interactions between users and peer support providers for a given post, unlike some other online support communities that allow multiple rounds of exchanges amongst users. Moreover, the forum involves anonymized posting of concerns, and the responses of trained peer support providers are moderated by a panel of mental health professionals before these are posted. These structural features may have influenced both the nature of posts received and the underlying purposes and expectations of the users. In other words, the findings may vary depending on the characteristics of the platform itself. In addition, several characteristics of the participants, such as education level, prior help-seeking, and other demographic or contextual factors, were not known, as these are not solicited on the forum to minimize respondent hesitancy and burden. This places limits on understanding the generalizability of the findings.

Despite these limitations, the study offers several implications for research and practice. Future work should examine whether such forums can function not only as spaces for strengthening self-help and peer support processes but also as avenues for improving professional help-seeking. Training peer support providers in ways that align with user needs, while simultaneously enhancing mental health literacy and reducing stigma and other barriers to professional help-seeking, is critical [53]. The nature of responses by peer support providers is likely shaped by their understanding of the purpose of posts and their inferences about the context and concerns expressed. Accordingly, the present findings can inform structured peer training to enhance responsiveness and contextual sensitivity. Training should emphasize recognizing markers of clinical severity; validating distress in relational narratives without the risk of escalating conflict; avoiding over-normalization of serious concerns; balancing emotional validation with nudging towards professional help-seeking as and when appropriate; and encouraging active collaboration and dialogue with their clinicians when treatment-related concerns are raised. These suggestions are grounded in patterns observed in the forum data.

## 5. Conclusions

Overall, the findings suggest that anonymized online support forums such as LTL can function as important safe spaces where individuals can openly articulate a wide spectrum of concerns, challenges, and struggles affecting their mental health and wellbeing. The predominant use of these platforms appears to center on disclosure of difficult emotions and situations, alongside processes of sense-making through peer sharing, validation, and seeking informal suggestions. While posts frequently referenced mood- and anxiety-related symptoms and experiences of significant distress, explicit queries or requests for recommendations regarding professional help-seeking were notably infrequent. These patterns underscore the role of such forums in fostering peer-based support and emotional expression, while also highlighting potential gaps in pathways to formal mental health care. The findings suggest that such forums may provide opportunities to reduce barriers to professional help-seeking, underscoring the need for further research in this area.

## Figures and Tables

**Table 1:** Themes and sub-themes in forum posts.

Sl. No.	Theme	Brief Description	Sub-Themes
1	Family (*n* = 17)	Influence of family relationships and expectations on wellbeing.	Sibling comparison, family conflict, wish to escape, role burden (non-normative), lack of family support, family pressure
2	Romantic Relationship (*n* = 14)	Challenges and emotions in romantic relationships.	Ambivalence, preoccupation, coping with breakup, jealousy
3	Concerns related to significant others (*n* = 4)	Worries about and challenges in supporting others’ mental health.	Concerns about the mental health of significant others, worries about not being helpful, challenges in supporting others
4	Feeling states (*n* = 84)	Varied negative emotional experiences and mood states.	Betrayal, stress, uncertainty, feeling stuck, worthlessness, hopelessness, death wishes, helplessness, desperation, rejection, numbness, loneliness, regret, anger, unfairness, burden, confusion
5	Mental health concerns (*n* = 23)	Specific symptoms and mental health diagnoses.	Depression, anxiety, social anxiety, phobia, schizophrenia, ADHD, memory issues, digital addiction, pornography addiction, trauma
6	Self-related concerns (*n* = 33)	Issues with self-esteem and self-perception.	Self-doubt, low self-confidence, shame, self-hatred, self-criticality
7	Concerns with implementation and planning (*n* = 11)	Difficulties in planning and managing responsibilities.	Time management, financial management

**Table 2:** Additional themes: description and excerpts.

Theme	Brief Description	Illustrative Excerpts
Feeling misunderstood/not understood (*n* = 9)	Users’ perceptions that their intentions, words, or emotions are not accurately perceived or acknowledged by others.	*“I constantly strive to do my best and of course as a human, I will fail at times, we all do. But for a strange reason she [mother] does not understand that…what I’m going through no matter how much I try to convey it to her”* (Post 1, Female, 19 years)
Unmet need for social approval (*n*= 8)	Users feel their presence, contributions, or opinions are overlooked or dismissed in social settings.	*“Sometimes I really feel left out by my surroundings, wherein after putting my 100% effort in some kind of event or situation no one would notice that…”* (Post 77, Female, 18 years)
Social comparison (*n* = 8)	Users evaluate their progress or abilities against peers and social norms.	*“Feeling like I have no talent in me And I can achieve nothing. I see that people of my age And younger are so talented and then there’s me.”* (Post 14, Female, 19 years)
Grief/loss (*n* = 3)	Users articulate emotional challenges such as sadness, loneliness after loss.	*“I am not able to accept it. And a part of me doesn’t want to move on as I really loved my partner.”* (Post 17, Female, 25 years)
Reflection and growth (*n* = 17)	Users describe concerns and self-reflection, indicating personal growth processes.	*“Lately I’ve realised that a lot of the things I believed about myself are not true. On one hand I am glad that i have the realisation but I’ve been also apprehensive about what next, now that I know this, how do I be a more genuine, honest version of myself.”* (Post 30, Male, 26 years)
Difficulty managing negative emotions (*n* = 5)	Users struggle to understand and navigate negative emotions and thoughts.	*“I got a lot of pent up emotions due to my past failures in competitive exams. I have this prejudice that I’m not good enough and that I won’t end up doing anything in life… I need specific help in letting go of my bottled up emotions. How do I become more emotionally open to myself?”* (Post 34, Male, 21 years)
Confusion about changes in self/mood (*n* = 7)	Users express confusion about noticeable shifts in emotions, preferences, or relationships.	*“I am really confused. I am not able to understand my feelings…I am not able to express my feelings to others.”* (Post 107, Female, 18) *“Its too hard to push myself. All the things that I could do energetically earlier, I have to try very hard to start. This is happening out of the blue”* (Post 51, Female)
Academic pressure (*n* = 14)	Users describe anxiety related to exams, performance, adjusting to changes in plans, and balancing academics with other things.	*“I have an intense form of performance anxiety when it comes to exams and tests. I usually pop a benzodiazepine before entering the exam hall to calm my nerves”* (Post 50, Male, 20 years)
Career challenges (*n* = 4)	Users share concerns about transitioning to work life, including life and loneliness due to relocation.	*“I’m going to graduate from college very soon and most (almost all) of my friends are moving to different cities for work…and I worry I’ll be all alone…I’m also worried about work…”* (Post 61, Male, 34 years)
Dissatisfaction with current state (*n* = 5)	Users describe stagnation, self-criticism, and lack of motivation in their current lives.	*“I have a weird feeling that I am lagging behind. I have taken a one year break to prepare for medical entrance at home. I see my school friends having fun in their college and here I am studying the same old thing. I neither feel anxious nor motivated to study. I feel I am just existing. Not feeling anything anymore!”* (Post 49, Male, 26 years)
Balancing relationships (*n* = 2)	Users discuss challenges in managing friendships, love, and family relationships.	*“It’s a constant struggle juggling with friendships, love and family. While all 3 seem to be the same yet different when I approach.”* (Post 48, Male, 24 years)
Efforts not leading to outcomes (*n* = 9)	Users describe frustration and disappointment when efforts do not yield desired results.	*“Despite the hard work, I have done nothing that seems to work. I did clear my previous backlogs but I still ended up getting one subject backlog”* (Post 7, Female, 19 years)

## Data Availability

The user posts analyzed in this study are publicly available on the Let’s Talk Life online peer support forum. The associated basic demographics (age and gender) and detailed coding data may be made available from the corresponding author upon reasonable request.
